# Frequency of Nucleated Red Blood Cells in the Peripheral Blood of ICU-Admitted Patients

**DOI:** 10.7759/cureus.33827

**Published:** 2023-01-16

**Authors:** Tayyab Noor, Ayisha Imran, Hassan Raza, Madiha Sarwar, Shereen Umer, Mavra Fatima

**Affiliations:** 1 Haematology, Chughtai Lab, Lahore, PAK

**Keywords:** icu death, frequency, edta vial, mortality, nucleated red blood cell, sepsis

## Abstract

Background

Nucleated red blood cells (NRBCs) are not normally found in the peripheral blood of normal healthy individuals. The presence of NRBCs on an adult peripheral blood smear indicates that there is an extremely high demand for the bone marrow to manufacture RBCs and that immature red blood cells are being released into the bloodstream. Anemia, myelofibrosis, thalassemia, miliary tuberculosis, malignancies of the bone marrow (myelomas, leukemias, lymphomas), and prolonged hypoxemia are a few possible pathogenic reasons. Critically ill patients who have NRBCs have a high mortality rate and a worse prognosis.

Objective: To evaluate the clinical significance of NRBCs in the peripheral blood of critically ill patients admitted to the ICU to find a cut-off to predict mortality.

Materials and Methods: A cross-sectional study was carried out over a period of six months September 1, 2020, to March 31, 2021, in Lahore, Pakistan. A total of 800 critically ill patients of both sexes in the age group of 18-70 years were included. Patients younger than 18 years and patients who underwent surgery were excluded. A quantity of 3 ml of whole blood sample in an ethylenediamine tetraacetic acid (EDTA) vial from each patient was run on SYSMEX XN-9000 (Sysmex Corporation, Kobe, Hyogo, Japan) and the results were reviewed on peripheral smears.

Results: The incidence of NRBCs in ICU-admitted patients was 62.5% (500/800). The total number of NRBC-positive patients recovering after the treatment was 364 (72.8%). The overall mortality of NRBC-positive patients was 30% (150/500). It was significantly higher (p<0.001) than that of NRBC-negative patients (14%; 44/300). During treatment, the highest mortality rate was seen in patients due to malignancy (100%), followed by sepsis (58.8%). It was observed that the disease pattern and number of NRBCs were significantly different (p<0.001) among all disease groups. However, there was no statistically significant difference in NRBCs on the basis of gender (p >0.05). In our study, a cutoff of NRBCs of 2.50 showed a high risk of mortality with a sensitivity of 91%.

Conclusion: The presence of NRBCs may predict mortality in critically ill ICU-admitted patients. Their presence in the blood may be regarded as a marker of severity suggesting a high risk of ICU death.

## Introduction

Progenitor cells of the mammalian erythropoietic lineage are nucleated red blood cells (NRBCs) [1,2]. Immature erythrocytes, sometimes referred to as erythroblasts, are referred to as NRBCs [3]. Under normal physiologic circumstances, NRBCs are typically absent in the peripheral blood of healthy persons [4]. Only the peripheral blood of newborns and premature infants exhibits tiny numbers (i.e., 0.5×10^9 /L) physiologically [5]. Stained peripheral blood smears were used to figure out the amount in the majority of previous studies on NRBCs; however, concentrations below 200/ul are challenging to find with this method [6]. Mechanized blood analyzers have been in use for a long time and offer a more practical and accurate method. NRBC concentrations of less than 100/ ul may frequently be measured with such an analyzer [7].

The emergence of these cells in the blood circulation can be triggered by specific pathological conditions and serious illnesses such as acute and chronic anemia, malignancy, congestive cardiac failure, and other hematological ailments. Many studies have also suggested that patients whose peripheral blood contains NRBCs have a poor prognosis [8]. The presence of erythropoietin, interleukin-3, and interleukin-6 in the peripheral blood has been linked to hypoxemia or infection in critical patients, indicating a decrease in tissue oxidation and/or inflammation [9]. NRBCs are a sign of both illness severity and an elevated risk of death in some patient populations, regardless of the etiology [10-12].In the current study, we estimated the frequency of NRBCs found in the blood of patients admitted to the intensive care unit (ICU) and its association with mortality in these subjects.

## Materials and methods

All ICU-admitted patients who received treatment between October 1, 2020, and March 31, 2021 (n =800 with 500 representing the NRBC-positive subset) from different departments at the National Hospital and Medical Centre, Lahore, Pakistan, were included in the study.

In this study, of the 500 NRBC-positive critically ill patients, 274 were males and 226 were females with ages ranging from 18 to 70 years. Patients under the age of 18 years and those who were recovering from surgery were excluded. The mean length of treatment in the ICU was 7 ± 1 days. A form requesting their voluntary and informed consent was signed by each patient in the trial. Chughtai Institute of Pathology's (CIP) Ethical Committee, Lahore, Pakistan, provided approval for the study (approval number: CIP/IRB/1050).

A quantity of 2 ml of whole blood from every patient was screened daily using an SYSMEX XN-9000 (Sysmex Corporation, Kobe, Hyogo, Japan) at CIP, following a method of measurement called fluorescence flow cytometry to determine the significance of NRBCs in the peripheral blood of critical care patients. Anticoagulated ethylenediamine tetraacetic acid blood samples were obtained from patients at least once daily until discharge from the ICU. The samples were not subjected to storage and old/degenerated samples were excluded. When NRBCs were found in the blood at least once, a patient was considered to be NRBC-positive for statistical purposes. Counts were expressed in NRBC cells (n)/l. If there were multiple NRBC measurements performed each day, the highest value was considered for the analysis. The results were then analyzed on peripheral smears.

Data were presented as the mean ± standard error of the mean. When samples were normally distributed, the differences between the data for deceased patients along with the mean number of NRBCs were analyzed using the one-way ANOVA. Significant level was determined by the Duncan test. A p-value of less than 0.05 was considered to be statistically significant. All calculations were performed by IBM SPSS Statistics for Windows, Version 23.0 (Released 2015; IBM Corp., Armonk, New York, United States).

## Results

**Figure 1:**
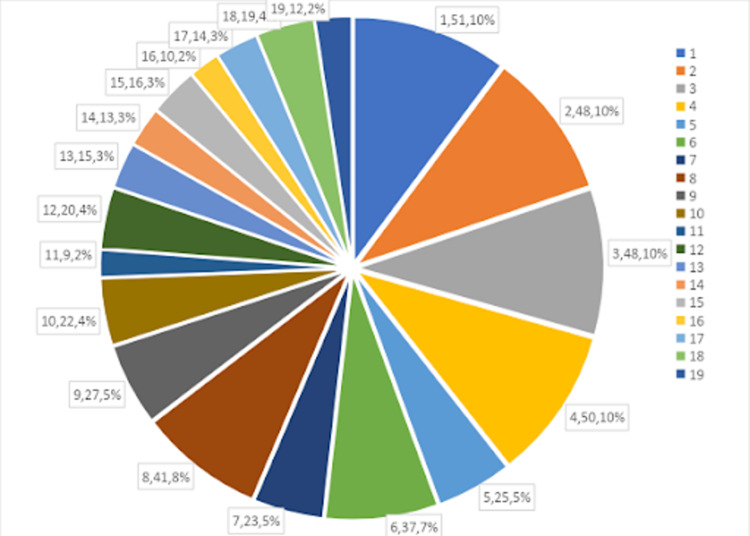
Distribution of NRBC-positive patients in different disease groups (n=500) 1: sepsis, 2: malignancy, 3: acute respiratory distress syndrome, 4: COVID-19 positive, 5: pulmonary embolism/hip fractures, 6: plasma cell myeloma, 7: beta-thalassemia, 8: fracture of femur, 9: postpartum hemorrhage/disseminated intravascular coagulation, 10: Guillain-Barre Syndrome, 11: chest discomfort/peptic ulcer, 12: diabetic ketoacidosis, 13: chronic kidney disease, 14: thrombotic thrombocytopenic purpura/hemolytic uremic syndrome, 15: intracranial bleed, 16: HIV positive,17: rectal bleed, 18: pleural effusion, 19: post-myocardial infarction NRBC: nucleated red blood cell; COVID-19: coronavirus disease 2019

**Figure 2:**
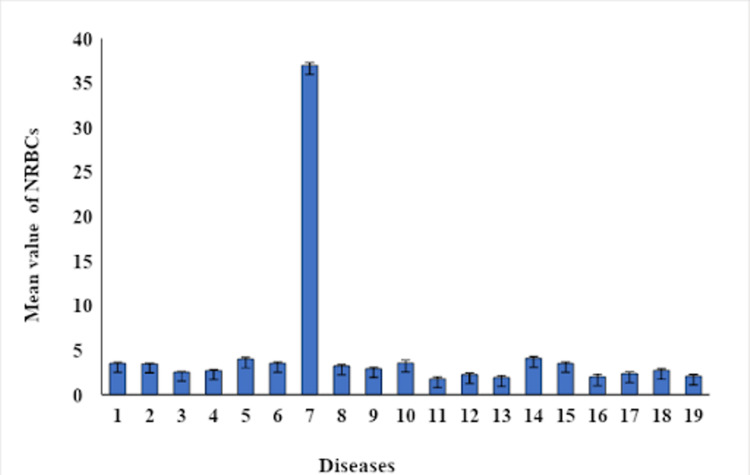
Number of NRBCs in different diseases Gender had no effect on NRBCs. Maximum NRBCs were observed in beta-thalassemia. 1: sepsis, 2: malignancy, 3: acute respiratory distress syndrome, 4: COVID-19 positive, 5: pulmonary embolism/hip fractures, 6: plasma cell myeloma, 7: beta-thalassemia, 8: fracture of femur, 9: postpartum hemorrhage/disseminated intravascular coagulation, 10: Guillain-Barre Syndrome, 11: chest discomfort/peptic ulcer, 12: diabetic ketoacidosis, 13: chronic kidney disease, 14: thrombotic thrombocytopenic purpura/hemolytic uremic syndrome, 15: intracranial bleed, 16: HIV positive,17: rectal bleed, 18: pleural effusion, 19: post-myocardial infarction NRBCs: nucleated red blood cells; COVID-19: coronavirus disease 2019

A total of 800 patients with ages ranging from 18 to 70 years were included in this study, out of which 500 patients were NRBC-positive (274 males, and 226 females). The incidence of NRBCs in ICU-admitted patients was 62.5% (500/800). The total number of NRBC-positive patients recovering after the treatment was 364 (72.8%) and 68.8% of these recovered patients showed no NRBCs. On average, NRBCs were detected 4±1 days following admission to the ICU but the trend was not noted and only monitored to assess treatment efficacy. The mean NRBC in different disease groups is given in Table 1. The overall mortality of NRBC-positive patients was 30% (150/500). It was significantly higher (p<0.001) than that of NRBC-negative patients (14% {44/300}). During treatment, the highest mortality rate was seen in patients with malignancy (100%), followed by sepsis (58.8%), coronavirus disease 2019 (COVID-19) (40%), and acute respiratory distress syndrome (31%). A graphical representation of the distribution of NRBCs in different disease groups is given in Figure 1. The mean value of NRBCs in different disease groups is shown in Figure 2. It was observed that the disease pattern and number of NRBCs were significantly different (p<0.001) among all disease groups. However, there was no statistically significant difference in NRBCs on the basis of gender (p >0.05). Maximum NRBCs were observed in beta-thalassemia patients (n=37).

**Table 1:** Different Diseases and Mean NRBCs NRBC: nucleated red blood cells; COVID-19: coronavirus disease 2019

Sr.No	Diseases	No. of patients	Mean No. of NRBCs
1	Sepsis	51	4
2	Malignancy	48	3
3	Acute Respiratory Distress Syndrome	48	3
4	COVID-19	50	3
5	Pulmonary Embolism/ Hip fracture	25	4
6	Plasma Cell Myeloma	37	4
7	Beta Thalassemia	23	37
8	Fracture Femur	41	3
9	Postpartum Hemorrhage (PPH)/ Disseminated Intravascular Coagulation (DIC)	27	3
10	Guillain-Barre Syndrome	22	4
11	Chest Discomfort	9	2
12	Diabetic Ketoacidosis (DKA)	20	2
13	Thrombotic Thrombocytopenic Perpura/ Hemolytic Uremic Syndrome	15	2
14	Intracranial Hemorrhage	13	4
15	HIV Positive	16	4
16	Rectal Bleed	10	2
17	Pleural Effusion	14	2
18	Post Myocardial Infraction	19	3
19	Stroke	12	2

**Figure 3:**
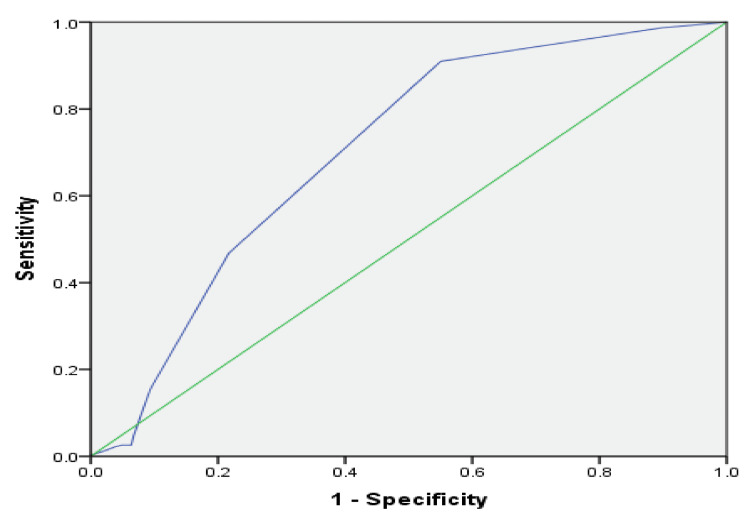
ROC curve to find out cut-off of NRBCs to predict mortality ROC: receiver operating characteristic; NRBCs: nucleated red blood cells

**Figure 4:**
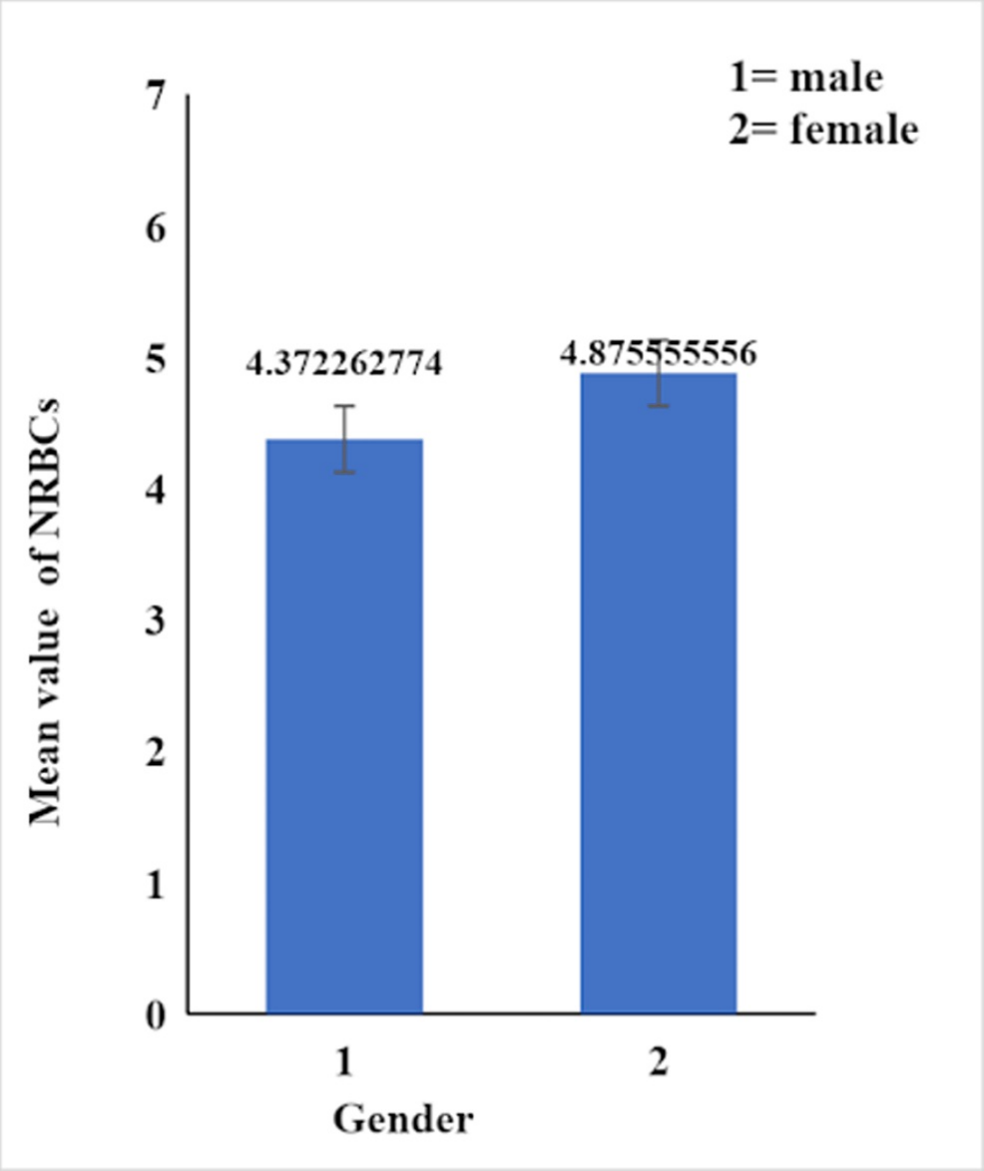
Mean NRBCs in male and female study participants NRBCs: nucleated red blood cells

The receiver operating characteristic (ROC) curve, which is defined as a plot of test sensitivity as the y-coordinate versus its 1-specificity or false positive rate (FPR) as the x-coordinate, is an effective method of evaluating the performance of diagnostic tests. An optimum cut-off serves as a meaningful tool to prognosticate patients, therefore we plotted a receiver operating characteristic (ROC) curve to find out the cut-off value of NRBCs to predict mortality. In our study, the best cutoff point for of NRBCs was 2.50 showing a high risk of mortality with a sensitivity of 91% and 1-specificity (false positive rate) of 55%, area under curve (AUC) =0.73 (Figure 3). Different numbers of NRBCs were observed in both genders due to different diseases and mean values are given in Figure 4 (p-value>0.05). 

## Discussion

Immature red blood cells are referred to as NRBCs and their presence in the bloodstream could be a sign of major issues with bone marrow function or RBC synthesis. A crucial component of laboratory hematology is the accuracy of NRBC enumeration, and this parameter should now be considered a potent aid in making decisions about diagnosis, prognosis, and treatment [13,14]. In previous studies and in the current one, it is demonstrated that the presence of NRBCs is linked to a prognosis that is generally not favorable [7].

The presence of NRBCs in peripheral blood has been shown to be an independent risk factor for increased mortality rates in ICU-admitted patients. Our study correlates with the findings of Shah and his colleagues who stated that NRBCs found in the peripheral blood of ICU patients are associated with an increased rate of mortality [10]. In our study, possible triggering factors behind higher mortality among patients having NRBCs were sepsis, malignancy, acute respiratory distress syndrome, COVID-19, deep venous thrombosis/pulmonary embolism, myocardial infarction, stroke, diabetic ketoacidosis, thrombotic thrombocytopenic purpura/hemolytic uremic syndrome (TTP/HUS), and HIV and AIDS. Furthermore, the results of the current investigation demonstrated that the finding of NRBC presence frequently occurs quite soon before death. Stachon et al. also reported that NRBCs were found in patients who died earlier in the disease course. NRBCs would therefore appear to be a warning sign of elevated danger [7]. Uncertainties exist regarding the pathophysiology of NRBCs in blood. Some researchers have claimed that hypoxemia, acute and chronic anemia, or severe infections are associated with the production of NRBCs in critically ill patients [7].

Hypoxic conditions (lower oxygen supply to tissues) stimulate the synthesis of red blood cells, which results in the presence of NRBCs in the circulation. Hemorrhage, anemia (hemolytic anemia, iron deficiency anemia, megaloblastic anemia), thalassemia major, severe lung illness, and congestive heart failure are some of these conditions [15]. In our study, hypoxia was found to be one of the major causes for the production of NRBCS in ICU-admitted patients. On the day of admission, a mean of three NRBCs per 100 WBCs was observed in patients of both genders. These patients were monitored on daily basis by screening their blood; however, the data were not categorized, and only the values that we obtained at the time of admission were statistically analyzed. Multivariate analysis of data in our study showed that different disease groups had significantly different numbers of NRBCs.

In the current investigation, hospitalized COVID-19-positive patients had worse outcomes and showed symptoms of hemopoietic stress. Fifty COVID-19 patients were admitted in critical condition and the majority (n=20; 40%) expired toward the end of the study. The blood-bone marrow barrier can be damaged by diseases that affect the bone marrow, allowing NRBCs to leak into the bloodstream [16]. Blood cancers (such as preleukemia, leukemia, lymphoma, multiple myeloma, myelofibrosis, and myelodysplasia), Neuroblastoma (cancer of developing nerve cells), beta-thalassemia, sarcoidosis (inflammation in the lungs, skin, eyes, and lymph nodes) [17], collagen vascular diseases (such as lupus), hemolytic uremic syndrome, myocardial infarction, and pulmonary embolism include such conditions. In our study, 51 patients suffering from sepsis were admitted to the ICU with an average of four NRBCs observed in them and all were critically ill. The majority of them recovered after treatment; however, 58.8% expired during the disease course. Kochhar et al.'s investigations also correlate with our findings [18]. Similarly in our study, patients suffering from stroke, post-myocardial infraction, pleural effusion, rectal bleeding, HIV, TTP/HUS, diabetic ketoacidosis, Guillain-Barre syndrome, and disseminated intravascular coagulation also had high levels of NRBCs in their blood. Kallen et al., similar to our study, reported that malignancy is also a cause of NRBC production and elevation [19]. In our study, 48 malignancy patients were brought to the ICU. All of them were expired and exhibited elevated levels of NRBCs. 

In our study, according to ROC curve analysis performed for the prediction of all-cause mortality, the best cut-off point for NRBC was 2.50. Many studies were conducted recently to find out the cut-off of NRBCs to predict mortality in different disease groups and different values were obtained. Narci et al. labeled NRBCs as a predictor of all-cause mortality in the emergency department and estimated a cut-off of >0 /μl (sensitivity 94,12%, specificity 82,35%, AUC =0.97) [20]. Cremer et al. showed that the optimal cut-off value of NRBCs for the prediction of death in low birth weight infants was > 2/nL with a sensitivity of 85% and a specificity of 75% [21]. Another study published that NRBC value at ICU admission was found to be an independent risk factor for mortality (OR 3.25; 95%CI 1.09-9.73, p = 0.035) and a cut-off level of 220 NRBC/µl was associated with more than tripled risk of ICU death (OR 3.2; 95% CI 1.93-5.35; p < 0.0001) [2].

Limitations

Because of the limited sample size and the fluctuation in NRBC count, this scientific study also has certain limitations. Moreover, old and degenerated samples or samples with NRBCs less than 200 ul will not be detected by the analyzer and will give false negative results. High white blood cell count may also contribute to false negative results.

## Conclusions

Immature red blood cells are referred to as NRBCs and their presence in the bloodstream could be a sign of major issues with bone marrow function or RBC synthesis. Although the number of NRBCs is not the same in different disease processes, the presence of NRBCs was associated with a higher mortality rate in critically ill ICU-admitted patients using an optimum cut-off value of 2.50. The overall mortality of NRBC-positive patients was significantly higher than that of NRBC-negative patients. During treatment, the highest mortality rate was seen in patients with malignancy followed by sepsis. A crucial component of laboratory hematology is the accuracy of NRBC enumeration, and this parameter should now be considered a potent aid in making decisions about diagnosis, prognosis, and treatment. NRBCs may predict mortality in ICU-admitted patients with high prognostic power. The presence of NRBCs in peripheral blood can be highlighted as a marker of disease severity and indicate a higher risk of ICU death. Estimation of NRBCs earlier during the hospital stay and risk stratification of patients can help in decreasing the mortality rate of patients and improve disease prognosis.
